# Admission Risk Score to Predict Inpatient Pediatric Mortality at Four Public Hospitals in Uganda

**DOI:** 10.1371/journal.pone.0133950

**Published:** 2015-07-28

**Authors:** Arthur Mpimbaza, David Sears, Asadu Sserwanga, Ruth Kigozi, Denis Rubahika, Adam Nadler, Adoke Yeka, Grant Dorsey

**Affiliations:** 1 Child Health & Development Centre, Makerere University, College of Health Sciences, Kampala, Uganda; 2 Infectious Diseases Research Collaboration, Kampala, Uganda; 3 Department of Medicine, San Francisco General Hospital, University of California San Francisco, San Francisco, United States of America; 4 National Malaria Control Program, Ministry of Health Uganda, Kampala, Uganda; 5 School of Public Health, Makerere University, College of Health Sciences, Kampala, Uganda; University of Louisville, UNITED STATES

## Abstract

Mortality rates among hospitalized children in many government hospitals in sub-Saharan Africa are high. Pediatric emergency services in these hospitals are often sub-optimal. Timely recognition of critically ill children on arrival is key to improving service delivery. We present a simple risk score to predict inpatient mortality among hospitalized children. Between April 2010 and June 2011, the Uganda Malaria Surveillance Project (UMSP), in collaboration with the National Malaria Control Program (NMCP), set up an enhanced sentinel site malaria surveillance program for children hospitalized at four public hospitals in different districts: Tororo, Apac, Jinja and Mubende. Clinical data collected through March 2013, representing 50249 admissions were used to develop a mortality risk score (derivation data set). One year of data collected subsequently from the same hospitals, representing 20406 admissions, were used to prospectively validate the performance of the risk score (validation data set). Using a backward selection approach, 13 out of 25 clinical parameters recognizable on initial presentation, were selected for inclusion in a final logistic regression prediction model. The presence of individual parameters was awarded a score of either 1 or 2 based on regression coefficients. For each individual patient, a composite risk score was generated. The risk score was further categorized into three categories; low, medium, and high. Patient characteristics were comparable in both data sets. Measures of performance for the risk score included the receiver operating characteristics curves and the area under the curve (AUC), both demonstrating good and comparable ability to predict deathusing both the derivation (AUC =0.76) and validation dataset (AUC =0.74). Using the derivation and validation datasets, the mortality rates in each risk category were as follows: low risk (0.8% vs. 0.7%), moderate risk (3.5% vs. 3.2%), and high risk (16.5% vs. 12.6%), respectively. Our analysis resulted in development of a risk score that ably predicted mortality risk among hospitalized children. While validation studies are needed, this approach could be used to improve existing triage systems.

## Introduction

Mortality rates among children admitted to many government hospitals in sub-Saharan Africa are high, ranging between 8–21%[1–6]. Most of these deaths occur within 24 hours of admission, and are attributable to preventable and treatable diseases, including pneumonia, diarrhea, malnutrition and malaria[7,8]. Reports from some health facilities suggest that high inpatient mortality rates may be partly attributable to poor case management[9,10]. However, despite improvements in the understanding of advanced pediatric care in resource limited settings, implementation of proven strategies has been difficult [11]. Limitations in resources and poor health-care systems compromise quality of health service delivery to critically ill children whose survival is dependent on timely and accurate medical attention.

Most government hospitals in sub-Saharan Africa attend to large numbers of children presenting with varying degrees of illness severity amidst constrained resources, uncoordinated systems of operation, and limited space compromising the quality of case management [12,13]. It is in such settings that triage, the timely and correct disposition of patients to priority groups for treatment, is a requirement to the provision of quality emergency service [14–16]. Triage systems, however, are non-existent or inadequate in most of these health facilities [17], likely contributing to avoidable hospital deaths [12,18].

The World Health Organization (WHO) advocates the use of the Emergency Triage Assessment and Treatment (ETAT) model to triage sick children in resource limited settings where staff are few with minimal equipment [19]. Implementation of ETAT and a modified ETAT model (ETAT+) is widely accepted as a centre piece for delivering quality pediatric hospital care in developing countries, and has been shown to improve the quality of emergency care in sub-Saharan Africa resulting in reductions in inpatient child mortality rates [1,2,20,21]. While ETAT is a powerful tool, it does have limitations. For one, ETAT has been difficult to implement and uptake has been poor following training programs suggesting that simplification of triage processes may achieve greater benefits[1,22,23]. Additionally, ETAT may not be the ideal triage system across all low resource health facilities [11,16]. Furthermore, ETAT is limited to assessment at admissionand does not provide for monitoring and triaging during hospitalization, yet patients may deteriorate during the course of hospitalization [24]. Data are needed to inform ways in which triage could be improved in low resource settings.

Here, we present a risk score for predicting inpatient pediatric mortality and informing triage decisions. The risk score is derived from data collected from 50249 pediatric admissions—including 1742 deaths—at four low-resource public hospitals in Uganda. These hospitals are part of a comprehensive inpatient malaria surveillance program that utilizes a standardized medical record from to capture patient data. Using a predictive analytical model, we developed a simple risk score based on easily recognisable clinical parameters used to categorize children into three different mortality risk groups, thus allowing for prioritization of care according to the risk of inpatient mortality at admission or during the course of hospitalization. Data collected one year prospectively at each hospital was used to validate the risk score.

## Materials and Methods

### Inpatient sentinel site surveillance system

**Fig 1:**
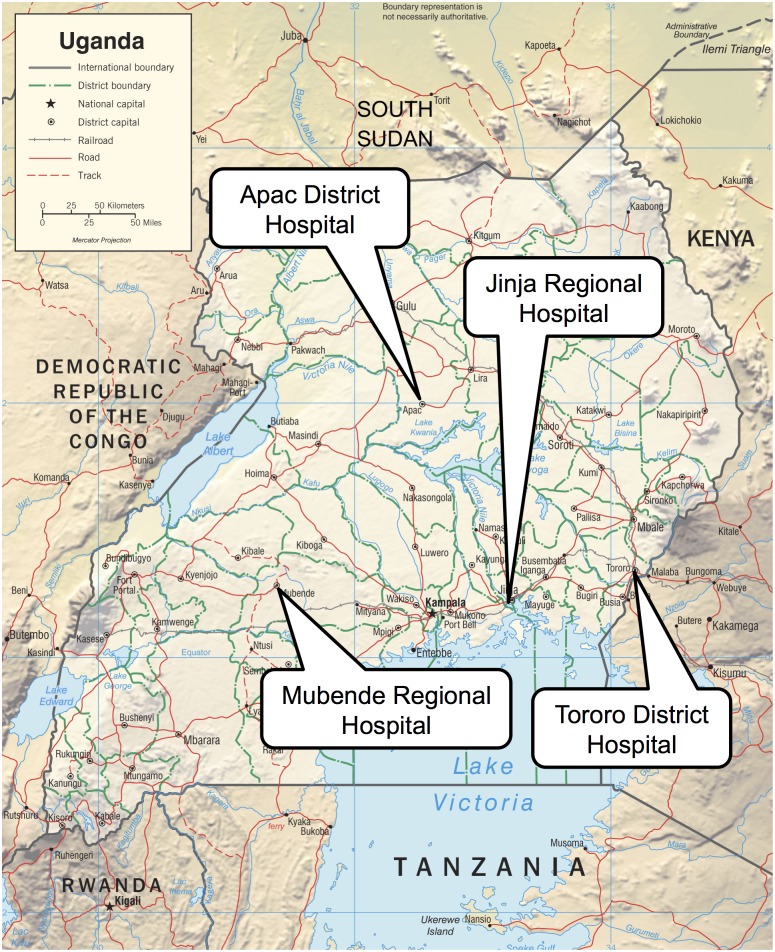
Study sites. Image adapted from the original (source: https://www.cia.gov/library/publications/cia-maps-publications/map-downloads/uganda-physiog.jpg/image.jpg).

Between April 2010 and June 2011, the Uganda Malaria Surveillance Project (UMSP) in collaboration with the National Malaria Control Program (NMCP) set up an enhanced sentinel site malaria surveillance program for children including neonates admitted to four public hospitals in different districts: Tororo, Apac, Jinja and Mubende (Fig 1). Tororo and Apac are general district hospitals providing basic inpatient health services. Jinja is a regional referral hospital offering specialist medical and surgical service. Mubende was elevated to a regional referral hospital one year after implementation of the surveillance program.

Due to the poor quality of Health Management Information System (HMIS) data, UMSP and the NMCP developed standardized methods for the collection of individual patient-level information with the aim of accurately monitoring trends in malaria burden, treatment practices, and clinical outcomes. Key among changes was the introduction of a standardized Medical Record Form (MRF) at all hospitals for the systematic documentation of patient details, including presenting symptoms and signs, laboratory test results, admission and final diagnosis, treatment administered and final outcome upon discharge. The MRF was completed by the clinician during each patient’s hospitalization and, where applicable, check boxes were used to minimize transcription errors and omission of vital information pertaining to the presenting illness. Additionally, the symptom and sign checklist adopted Integrated Management of Childhood Illness (IMCI) terminologies, a standard framework used for patient management in Uganda which most clinicians are familiar with. This system maximized opportunities for capture of patient information.

The UMSP sentinel site surveillance system is funded by the Centers for Disease Control and Prevention (CDC). As this system collects routine health information to supplement HMIS and all patient data are anonymized and de-identified prior to entry, it is considered to be nonresearch per the CDC Policy “Distinguishing Public Health Research and Public Health Nonresearch” [25]. Institutional Review Board approval and informed consent was not deemed necessary for this analysis.

### Training

Prior to starting surveillance, hospital staff received training, sensitizing them to the importance of quality data in relation to malaria surveillance. Clinical staff, comprised of medical officers, clinical officers, nurses, and midwives, were introduced to the new MRF. Training emphasized the importance of good medical record-keeping, including documentation of patient information pertaining to illness, and fostering a culture of utilizing data to improve the quality of services.

### Patient care

Management of children in the hospital was primarily the duty of clinical officers (diploma level training in medicine) and nurses in Tororo, Apac and Mubende hospitals. Typically, children were seen by a clinical officer in the outpatient department, who upon assessment, directed an initial plan of management including tests to be done, decision to admit the patient and need of emergency care. While in the ward, nurses reviewed children, and occasionally a clinical officer or a medical doctor was called in to review critically ill children. Staffing at Jinja Hospital differed from the other hospitals. In addition to clinical officers, intern doctors and three pediatricians were responsible for assessing and reviewing children on a daily basis, and at least one pediatrician was available each day of the week, including weekends.

### Quality assurance

To maintain standards, routine supervisory visits were conducted on a quarterly basis by the UMSP surveillance team consisting of a physician, laboratory supervisor, and data supervisor. During visits, data quality was assessed and feedback provided. UMSP took responsibility of printing and supplying the MRF to all four hospitals. In liaison with the district health office, UMSP supported all site laboratories, ensuring adequate staffing, coverage and adherence to high standards of malaria microscopy at participating hospital laboratories. In addition, a back-up system guaranteeing continuous supply of reagents and slides was maintained.

### Data management

MRFs of discharged patients were collected on a daily basis for electronic data entry using Access (Microsoft Corporation, Redmond, WA). Each month, after all MRFs were entered, data were sent electronically to a central facility in Kampala, where data from sites were checked for errors. Once all queries were addressed, data from respective sites were merged into relational databases comprised of data from previous months.

### Data analysis

Statistical analysis was performed using Stata 12.0 (Stata Corp, College Station, TX) and was performed using two datasets. The first, ‘derivation dataset’ comprised of data collected from the date when the program started at each site (between April 2010 and June 2011) and concluding in March 2013, and was used to derive a logistic regression predictive model that was used to generate the risk score. The second, ‘validation dataset’ comprised of data collected subsequently from each site, starting in April 2013 up till March 2014and was used to prospectively validate the performance of the risk score.

### Risk score generation

The risk score was developed using the derivation dataset, and was based on consideration of 25 clinical parameters that could rapidly be assessed on initial presentation and for which data existed (S1 Appendix). Multivariable logistic regression was used to develop a predictive model for the association between predictor variables and the outcome of interest, inpatient mortality, defined as any child with a disposition marked as “death” on the MRF. Variables considered for inclusion in the predictive modelwere binary and included (S2 Appendix) age, gender, nine symptoms (fever, cough, difficulty breathing, convulsions, altered consciousness, vomiting, unable to drink or breastfeed, diarrhea, tea colored urine), and 14 physical exam findings (temperature, lethargy, unconsciousness, unable to sit, pallor, jaundice, deep breathing, intercostal retractions, subcostal retractions, stridor, wheezing, rhonchi, crackles, meningeal signs). Thus, starting with 25 variables and using backward selection, 12 variables were eliminated from the initial model on the basis of not being predictive (OR<1.0) or having a non-significant p-value (p >0.05) leaving 13 predictor variables in the final multivariate model. We utilized the regression coefficient of each predictor variable in the final model to generate the risk score. A value of ‘one’ or ‘two’ was awarded to each predictor variable based on the following cut-off; one when the regression coefficient was <1.0 and two when the regression coefficient was ≥1.0. A score of zero was given for any of the final 13 predictor variables for which data were missing. For each individual patient, values for each of the 13 predictor variables were summated and a composite risk score generated. With the aim of providing a simple and efficient potential triage system, we grouped the overall risk score into three categories; low, medium, and high, which was the final product of the derivation phase.

### Validating the performance of the risk score

To visualize the predictive value of the risk score in correctly classifying patients as deaths and non-deaths, receiver operator characteristic (ROC) curves for both the derivation and validation data sets were plotted. The area under the curve (AUC) for each ROC curve was calculated and results compared. To evaluate how well the risk categories predicted mortality, we calculated the predicted relative mortality risk in the moderate and high risk groups compared to the low risk group as the reference standard using both the derivation and validation data set. Predicted relative mortality risks were compared for consistency in performance across datasets and the sites.

## Results

### Study population and characteristics

The derivation data set comprised of 50249 admissions, of which 44981 (90%) admissions had no missing data on any one of the 13 variables included in the final predictive model. The validation data set comprised of 20406 admissions, of which 18779 (92%) had no missing data. Characteristics of patients were similar during both periods (Table 1). Overall, most children were under five years of age during the derivation (87.2%) and validation(83.9%) periods. A small proportion of the children were neonates in both the derivation (3.2%) and validation (3.1%) data sets. The median age was 18 months (IQR 9–36) in the derivation and 21 (IQR 10–38) in validation databases. The proportion of patients who died during their hospitalization was3.5% during the derivation and 2.7% validation periods.

**Table 1:** Characteristics of hospitalized patients.

Characteristics	Derivation period April 2010 –March 2013					Validation period April 2013 –March 2014				
	Hospital					Hospital				
	All	Tororo	Jinja	Mubende	Apac	All	Tororo	Jinja	Mubende	Apac
**Months of data collection**	36	36	31	24	22	12	12	12	12	12
**Total admissions**	50249	16383	22766	7049	4051	20406	5563	6742	4413	3688
**Females, n (%)**	22778 (45.3%)	7432 (45.3%)	10360 (45.5%)	3229 (45.8%)	1757 (43.3%)	9430 (46.3%)	2563 (46.4%)	3104 (46.0%)	1991 (45.1%)	1772 (48.0%)
**Children under 5 years, %**	87.2%	91.4%	86.3%	85.3%	77.9%	83.9%	87.7%	84.7%	83.3%	77.3%
**Age in months, median (IQR)** ^**a**^	18 (9–36)	15 (8–27)	18 (8–36)	18 (10–36)	24 (13–48)	21 (10–38)	18 (9–36)	18 (9–36)	20 (11–39)	29 (16–48)
**Days hospitalized, median (IQR)** ^**a**^	3 (2–4)	3 (2–4)	2 (2–4)	3 (2–5)	3 (2–5)	3 (2–5)	3 (2–4)	3 (2–5)	4 (2–6)	3 (2–4)
**Discharged alive, n (%)**	41907 (83.4%)	13748 (83.9%)	18782 (82.5%)	5680 (80.6%)	3697 (91.3%)	17236 (84.5%)	4843 (87.1%)	5283 (78.4%)	3764 (85.3%)	3346 (90.7%)
**Death, n (%)**	1742 (3.5%)	293 (1.8%)	1025 (4.5%)	324 (4.6%)	100 (2.5%)	556 (2.7%)	85 (1.5%)	326 (4.8%)	102 (2.3%)	43 (1.2%)
**Referred, n (%)**	527 (1.0%)	166 (1.0%)	79 (0.3%)	242 (3.4%)	40 (1.0%)	246 (1.2%)	63 (1.1%)	73 (1.1%)	76 (1.7%)	34 (0.9%)
**Absconded, n (%)**	6073 (12.1%)	2176 (13.3%)	2880 (12.7%)	803 (11.4%)	214 (5.3%)	2368 (11.6%)	572 (10.3%)	1060 (15.7%)	471 (10.7%)	265 (7.2%)

^**a**^Interquartile range

### The predictive model and generation of the risk score

Results of the predictive modelused in generating the risk score are presented in Table 2. Age≤ 4 months had the highest odds of inpatient mortality (3.43; 95% CI 3.00–3.92) and was the only variable with a regression coefficient > 1.0 and was assigned two points in the final risk score. The remaining 12 variables had regression coefficients ranging between >0 and < 1 and were each assigned a score of one, resulting in a cumulative risk score ranging from a minimum of zero to a maximum score of 14.

**Table 2:** Multivariate analysis used in deriving risk score.

Risk factor	Missing data	Prevalence	Risk of death	Odds ratio (95% CI)	Risk Score
**Symptoms**					
Age ≤ 4 months	8 (<0.1%)	5,001 (10.0%)	465 (9.3%)	3.43 (3.00–3.92)	2
No subjective fever	148 (0.3%)	4,230 (8.4%)	310 (7.3%)	2.39 (2.05–2.78)	1
Difficulty breathing	620 (1.2%)	8,073 (16.1%)	574 (7.1%)	1.51 (1.32–1.72)	1
Altered consciousness	692 (1.4%)	2,416 (4.8%)	303 (12.5%)	1.73 (1.42–2.10)	1
Unable to drink/breastfeed	601 (1.2%)	7,845 (15.6%)	548 (7.0%)	1.27 (1.11–1.46)	1
Convulsions	574 (1.1%)	7,144 (14.4%)	430 (6.2%)	1.38 (1.19–1.60)	1
**Signs**					
Temperature ≤ 35.5°C	3,781 (7.5%)	1,030 (2.0%)	112 (10.9%)	2.45 (1.95–3.09)	1
Pallor	1,017 (2.0%)	18,719 (37.3%)	866 (4.6%)	1.44 (1.28–1.62)	1
Jaundice	1,139 (2.3%)	1,154 (2.3%)	87 (7.5%)	1.88 (1.45–2.43)	1
Deep breathing	456 (0.9%)	10,386 (20.7%)	740 (7.1%)	1.55 (1.36–1.76)	1
Unconscious	678 (1.3%)	1,165 (2.3%)	242 (20.8%)	2.58 (2.07–3.20)	1
Unable to sit up or stand	469 (0.9%)	6,204 (12.5%)	558 (9.0%)	1.79 (1.54–2.08)	1
Signs of meningitis	615 (1.2%)	752 (1.5%)	98 (13.0%)	1.91 (1.46–2.50)	1

### Validating the performance of the risk score

**Fig 2:**
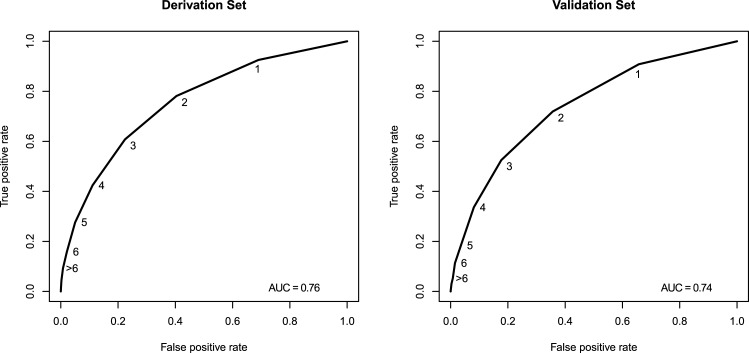
Receiver operator characteristic for both the derivation and validation data sets (including the “area under the curve (AUC)”). The infection points and labels on the curves represent the risk scores and represent the predictive value of the risk score.

**Fig 3:**
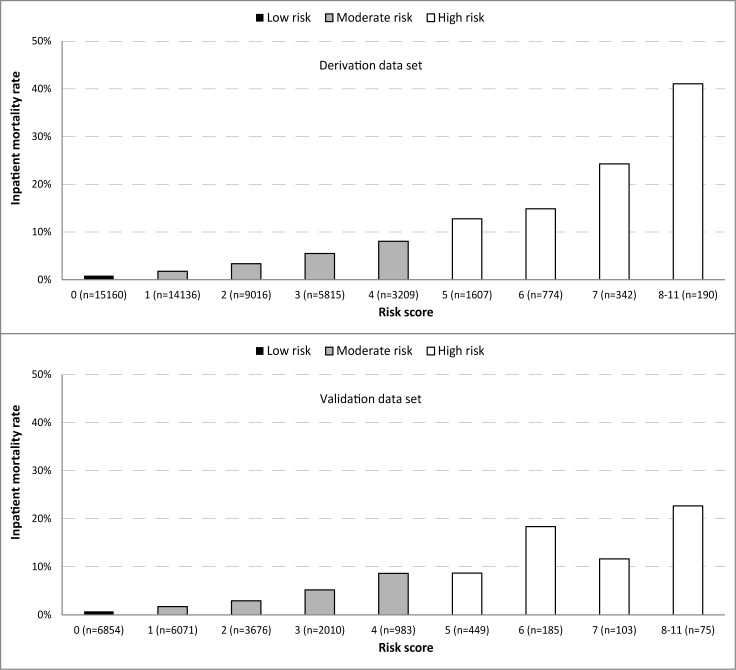
The predicted risk of mortality per unit increase in risk score categorized into three risk categories.

The receiver operator characteristic (ROC) curves (Fig 2)and associated AUC for the derivation (AUC 0.76) and validation (AUC 0.74)datasets demonstrated good levels of predictive performancein terms of accuratelyclassifying those who died and those who didn’t. As the risk score increased, so did the inpatient mortality rate,akin to a dose response curve (Fig 3). Using this curve we were able to transform the cumulative risk score into three categories, each category adequately discriminating the associated mortality rate. Those with a risk score of zero (n = 15160, 30%) had a mortality rate of 0.8% and were categorized as low risk. Those with a risk score of 1–4 (n = 32176, 64%) had a mortality rate of 3.5% and were categorized as moderate risk (Table 3). Children with a risk score of ≥ 5 (n = 2913, 6%) had a mortality rate of 16.5% and were categorized as high risk. When the same cutoffs where applied to the validation data set associated mortality rates; low risk (0.7%), moderate risk (3.2%), and high risk (12.6%) were comparable to those obtained using the derivation dataset.

**Table 3:** Associations between risk groups and mortality stratified by study site.

Study site	Low risk group*		Moderate risk group				High risk group			
	N	Mortality	N	Mortality	RR (95% CI)	p-value	N	Mortality	RR (95% CI)	p-value
**Derivation data set**										
All sites	15160	0.8%	32176	3.5%	4.09 (3.42–4.90)	<0.001	2913	16.5%	19.2 (15.9–23.2)	<0.001
Tororo	7488	0.7%	8625	2.4%	3.57 (2.63–4.86)	<0.001	270	13.7%	20.5 (13.6–30.8)	<0.001
Jinja	4213	1.4%	16626	4.0%	2.86 (2.19–3.73)	<0.001	1927	15.5%	11.0 (8.42–14.6)	<0.001
Mubende	1325	0.4%	5079	3.8%	10.1 (4.15–24.4)	<0.001	645	19.5%	51.7 (21.3–125.9)	<0.001
Apac	2134	0.7%	1846	3.5%	4.69 (2.72–8.08)	0.003	71	26.7%	35.7 (19.2–66.4)	<0.001
**Validation data set**										
All sites	6854	0.7%	12740	3.2%	4.25 (3.18–5.68)	<0.001	812	12.6%	16.9 (12.2–23.4)	<0.001
Tororo	2604	0.5%	2892	2.4%	5.10 (2.77–9.40)	<0.001	67	7.5%	16.2 (5.87–44.7)	<0.001
Jinja	1397	1.7%	5010	4.6%	2.67 (1.76–4.05)	<0.001	335	21.5%	12.5 (8.01–19.5)	<0.001
Mubende	960	0.4%	3095	2.5%	5.97 (2.19–16.3)	<0.001	358	5.9%	14.1 (4.87–40.7)	<0.001
Apac	1893	0.6%	1743	1.6%	2.76 (1.38–5.54)	0.003	52	7.7%	13.2 (4.36–40.2)	<0.001

* Reference group

The three risk categories adequately predicted mortality risk using both the derivation and validation datasets (Table 3). Using the validation dataset and low risk group as a reference, the overall predicted relative mortality risk were significant in the moderate risk group (RR 4.09; 95% CI 3.42–4.90) and in the high risk group (RR 19.2; 95% CI 15.9–23.2) with findings matching expectation and were consistent across sites. The predicted relative mortality risks were repeated using the validation dataset, and performance was comparable to that based on the derivation data set (Table 3). Of note, in Mubende and Apac the predicted relative risks of mortality in the moderate risk and high risk group were higher based on the derivation database compared to the validation database.

## Discussion

We developed a simple risk score for predicting mortality by categorizing patients into low, moderate, and high risk groups. The risk score was generated based on regression coefficients of a logistic regression predictive model. The performance of the risk score in terms of correctly predicting children who died and those who didn’t was found to be good even upon prospective validation with a temporal data set. Upon categorization of the risk score into, low, medium and high categories, the associated mortality risk was distinct and reflective of the assigned risk category, a pattern consistent across all sites. Furthermore, using the low risk category as the reference group, the predicted relative mortality risk in the moderate and high risk matched expectation, and was consistent across sites. Comparable levels of performance were replicated using the validation dataset.

Early recognition of serious illness may reduce risks of morbidity and mortality in sick children, highlighting the importance of improving triage services in resource-limited settings [26]. However, because most public health facilities in sub-Saharan Africa operate under extreme conditions of constraint [17], interventions designed to improve triage systems in these health facilities need to be simple and easily adaptable to existing conditions, yet maintain other qualities of an ideal triage scale. Risk scores used in the developed world, such as PRISM (Pediatric Risk of Mortality), MODS (Multi-Organ Dysfunction Score), and APACHE (Acute Physiologic and Chronic Health Evaluation) where designed for use in well equipped Intensive Care Units, relying on physiological parameters that are not routinely available in resource-limited settings [27]. The BCS (Blantyre Coma Scale) and a simplified version of MODS have been used in the developing world but investigations of both systems have largely been limited to patients with malaria, thus limiting their generalizability [28,29].

The ideal triage scale should be simple to understand and implement, rapidly applicable, have high rates of inter-rater agreement, facilitate appropriate placement, predict resource requirements and predict clinical outcome reflective of severity of illness [12]. The ETAT (Emergency Triage Assessment and Treatment) model, recommended for triage in resource-limited settings relies on rapidly evaluating the sick child for six signs that would require emergency treatment and 12 signs that would require priority treatment [26]. The approach is credited for being simple, and if well implemented has been shown to reduce inpatient child mortality rates in resource-limited settings at an affordable cost [1,30,31]. ETAT’s simplicity in approach, which is focused on not missing critically ill children, may however, lead to misclassification of children with mild illness as severely ill. This could result in wastage of resources and exposure of patients to unnecessary interventions, some of which are associated with harm, thus undermining the benefits of ETAT[22,32,33]. In addition, the ETAT model focuses more on appropriate placement of attention, with little regard to predicting clinical outcome, thus limiting its use in determining the best allocation of available medicine and in informing decision making when faced with a patient at high risk of death. Furthermore, the ETAT model only caters for initial assessment and does not provide for continued monitoring and re-evaluation of the patient.

The risk score presented here can inform triage decisions in important ways. For one, the scoring system is based on easily recognizable clinical criteria, factoring in both reported history and signs that can simply be elicited upon evaluation of the patient by low level health workers. In addition, the criteria do not include parameters that are laborious to measure or require expensive equipment, easing measurement. For pragmatic reasons, and with exception of one parameter that earned a score of 2, fulfillment of the remaining 12 parameters earned a score of 1, simplifying the process of generating a composite score for individual patients. We believe the proposed risk score offers a simple yet, objective and accurate means of predicting mortality risk and for prorating management decisions among hospitalized children offering opportunities for improving existing triage systems in resource-limited settings. The risk score has potential for use in monitoring patient’s progress during hospitalization informing management decisions. Indeed, a triage scale or scoring system should in addition to prognostication of outcomes, be of use in monitoring illness progression during hospitalisation, particularly in the setting of emergency care when dealing with unstable states. A scoring scale developing in Malawi for predicting mortality among children after hospital admission showed impressive levels of performance, but is limited by reliance on parameters that would not easily be measured in low resource settings[27].

Our study is not without limitations. The risk score was developed in hospitals serving as malaria sentinel sites, which may not be representative of a typical government hospital, limiting the external validity of the risk score. In addition, despite efforts at prospectively validating the performance of the risk score using temporal data, reliance on data collected from the same site may have biased the results favorably. Indeed, there remains uncertainty as to how well the risk score would perform prospectively and in settings where doctors are absent, and nurses, nursing aids and lower level cadres serve as frontline health workers, as is the reality in most government hospitals in developing countries. In that regard an independent prospective external validation using data collected from an appropriate (similar) patient population in a different site is recommended. Furthermore, missing data on some variables including pulse, respiratory rate, and measures of nutritional status, and HIV status limited opportunities of studying the influence of these important clinical measures on the predictor model and the risk score, potentially undermining the utility of the risk score in situations where diseases associated with these measures are prevalent. Inability to compare the performance of the risk score to ETAT is a major limitation considering that the latter is the recommended triage system in many countries in sub-Saharan Africa. Lastly, we were unable to account for the impact of in patient management on patient outcomes, particularly among children with malnutrition whose risk of death has been shown to increase when transfused with blood or given intravenous fluids without caution [34]. Considering that blood transfusion and intravenous fluid infusions are common in these settings, it is possible that this practice influenced mortality risk.

## Conclusions

Despite limitations, the risk score presented here can inform triage decisions in important ways. For one, the scoring system is based on easily recognizable clinical criteria, factoring in both reported history and signs elicited upon evaluation of the patient. For pragmatic reasons, and with the exception of one parameter that earned a score of 2, fulfillment of the remaining 12 parameters earned a score of 1, simplifying the process of generating a composite score for individual patients. We believe the proposed risk score offers an objective means of predicting mortality risk and for prorating management decisions among hospitalized children offering opportunities for improving existing triage systems in resource-limited settings. However, it remains unknown if rationalized of management and treatment decisions based on the risk score would actually impact on mortality. Indeed, if the performance of the risk score is proven to be worthy upon external validation in other settings, studies to evaluate the likely impact of the risk score on mortality are also warranted.

## Supporting Information

S1 Appendix Medical Record Form. (DOCX)

S2 Appendix Risk score generation. (DOCX)

S1 Dataset (ZIP)
