# Supplementary material for: Admission Risk Score to Predict Inpatient Pediatric Mortality at Four Public Hospitals in Uganda
Source: PLoS One. 2015 Jul 28;10(7):e0133950. doi: 10.1371/journal.pone.0133950 (PMC4517901; doi:10.1371/journal.pone.0133950)
Supplement: S1 Appendix — (DOCX) [file pone.0133950.s001.docx]

| **MOH-UMSP INPATIENT PAEDIATRIC SURVEILLANCE CASE RECORD FROM** | | | | | | | | |
| --- | --- | --- | --- | --- | --- | --- | --- | --- |
| **Date of admission** **:** \|___\|___\| **day** \|___\|___\| **month** \|___\|___\| **year** | | | | **In patients number: ____________________________________** | | | | |
| **Time of admission:** \|___\|___\| **hours** \|___\|___\| **min □ am □ pm** | | | | **Ward:** | | | **Bed number:** | |
| **Patient’s name: Last_____________________________First_______________________________________** | | | | | | | **Gender:** □ Male □ Female | |
| **Age:** \|___\|___\| **years** \|___\|___\| **months**  \|___\|___\| **days**  **( If less than 5 years) ( If less than 1 month)** | | | | **Re-admission (Admission of patient within 30days of discharge from same hospital) :** □ Yes □ No if yes, how many days: ________days | | | | |
| **ADDRESS AND NEXT OF KIN** | | | | | | | | |
| **Village LCI:** | | **Parish:** | | | | **Sub-county:** | | |
| **District:** | | **Name of LC1 chairman:** | | | | | | |
| **Name of NOK:** | | | | **Relationship with NOK:** | | | | |
| **Name of father (if not NOK)** | | | | **Telephone contact:** | | | | |
| **Religion:** | | | | **Tribe:** | | | | |
| **Referral from** | □ Self referral | | □ Health Centre IV | | □ Government hospital | | | □ Private hospital |
|  | □ Private clinic | | □ Other, if yes, specify details: | | | | | |

| **HISTORY/SYMPTOM CHECK LIST** | | | | | | | | | | | |
| --- | --- | --- | --- | --- | --- | --- | --- | --- | --- | --- | --- |
| **SYMPTOM**  **(all must be answered)** | | **PRESENT** | | **SYMPTOM**  **(all must be answered)** | | **PRESENT** | | **SYMPTOM**  **(all must be answered)** | | **PRESENT** | |
| **Fever** | | □ Yes | □ No | **Convulsions** | | □ Yes | □ No | **Diarrhoea** | | □ Yes | □ No |
| **Cough** | | □ Yes | □ No | **Altered consciousness** | | □ Yes | □ No | **Diarrhoea > 2 weeks** | | □ Yes | □ No |
| **Cough more than 2 weeks** | | □ Yes | □ No | **Vomiting everything** | | □ Yes | □ No | **Blood diarrhoea** | | □ Yes | □ No |
| **Difficulty in breathing** | | □ Yes | □ No | **Unable to drink/breastfeed** | | □ Yes | □ No | **Passing tea coloured urine** | | □ Yes | □ No |
| **Other history:** | | | | **Past medical history** | | | | **Feeding history** | | | |
| **Immunization details** | □ None | | | □ Complete | □ Incomplete | | | □ On schedule | □ Not known | | |

| **VITAL SIGNS** | | | | | | | | | | | | | | | | | | | | | |
| --- | --- | --- | --- | --- | --- | --- | --- | --- | --- | --- | --- | --- | --- | --- | --- | --- | --- | --- | --- | --- | --- |
| Temp: _____ °C | | | | | Weight: _____ / kg | | | | | | Pulse: _____ / min | | | | BP: _____ / ____ mmHg | | | | Respiratory rate: ____/min | | |
| **GENERAL EXAMINATION** | | | | | | | | | | | | | | | | | | | | | |
| **Pallor:** | □None | □Mild | | □ Mod | | | | □ Severe | | **Severe wasting:** | | □ Yes | | □ No | **Sunken eyes** | □ Yes | | □ No | | **MUAC** | _______cm |
| **Skin pinch return (s):** | | | □ 0 | | | □ 1 | | | □ 2 | **Edema:** | | □ Yes | | □ No | **Jaundice** | □ Yes | | □ No | | **Height** | _______cm |
| **RESPIRATORY SYSTEM** | | | | | | | | | | | | | **CARDIOVASCULAR SYSTEM** | | | | | | | | |
| **Deep breathing:** □ Yes □ No | | | | | | | **Airway:** □ Clear □ Strider | | | | | | **Pulse:** □ Normal □ Weak | | | | **Cap refill:** □ < 2s □ 2-3s □ > 3 | | | | |
| **Flaring of nostrils:** □ Yes □ No | | | | | | | **Wheezing:** □ Yes □ No | | | | | |  | | | | | | | | |
| **Intercostal recession:** □ Yes □ No | | | | | | | **Rhonchi:** □ Yes □ No | | | | | |  |  |  |  |  |  |  |  |  |
| **Subcostal recession:** □ Yes □ No | | | | | | | **Crackles:** □ Yes □ No | | | | | |  |  |  |  |  |  |  |  |  |
| **CENTRAL NERVOUS SYSTEM** | | | | | | | | | | | | | **RESPIRATORY SYSTEM** | | | | | | | | |
| **Unconscious:** □ Yes □ No | | | | | | | **Bulging fontanelle:** □ Yes □ No | | | | | |  | | | | | | | | |
| **Lethargy:** □ Yes □ No | | | | | | | **Stiff neck:** □ Yes □ No | | | | | |  |  |  |  |  |  |  |  |  |
| **Unable to sit/stand:** □ Yes □ No | | | | | | | **Kerning’s sign:** □ Yes □ No | | | | | |  |  |  |  |  |  |  |  |  |
| **BCS** ________ **Eye Opening:** Spontaneous (1); Does not follow light (0); **Motor response**: Localizes pain (2); Unable to localize pain (1); No movement (0); **Verbal response:** Normal cry (2); Weak cry (1); No sound (0) | | | | | | | | | | | | | | | | | | | | | |
