# Supplementary material for: Admission Risk Score to Predict Inpatient Pediatric Mortality at Four Public Hospitals in Uganda
Source: PLoS One. 2015 Jul 28;10(7):e0133950. doi: 10.1371/journal.pone.0133950 (PMC4517901; doi:10.1371/journal.pone.0133950)
Supplement: S2 Appendix — (DOCX) [file pone.0133950.s002.docx]

Appendix 2: Risk score generation using derivation dataset

Variables included in final risk model

Age

Symptoms: fever, difficulty breathing, altered consciousness, unable to drink or breastfeed, convulsions

Physical exam: temperature, unconsciousness, pallor, jaundice, deep breathing, meningeal signs, unable to sit up

Variables removed using backwards selection

Gender

Symptoms: cough, vomiting, diarrhea, tea colored urine

Physical exam: lethargy, intercostal retractions, subcostal retractions, stridor, wheezing, rhonchi, crackles

Initial variables

Age

Gender

Symptoms: fever, cough, difficulty breathing, convulsions, altered consciousness, vomiting, unable to drink or breastfeed, diarrhea, tea colored urine

Physical exam: temperature, lethargy, unconsciousness, unable to sit up, pallor, jaundice, deep breathing, intercostal retractions, subcostal retractions, stridor, wheezing, rhonchi, crackles, meningeal signs
